# Blood Pressure in relation to 24-Hour Urinary Sodium and Potassium Excretion in a Uruguayan Population Sample

**DOI:** 10.1155/2018/6956078

**Published:** 2018-12-02

**Authors:** Paula Moliterno, Ramón Álvarez-Vaz, Matias Pécora, Leonella Luzardo, Luciana Borgarello, Alicia Olascoaga, Carmen Marino, Oscar Noboa, Jan A. Staessen, José Boggia

**Affiliations:** ^1^Departamento de Nutrición Clínica, Escuela de Nutrición, Universidad de la República, Montevideo, Uruguay; ^2^Instituto de Estadística, Universidad de la República, Montevideo, Uruguay; ^3^Departamento de Fisiopatología, Universidad de la República, Montevideo, Uruguay; ^4^Centro de Nefrología, Universidad de la República, Montevideo, Uruguay; ^5^Laboratorio de Patología Clínica, Universidad de la República, Montevideo, Uruguay; ^6^Área de Investigación, Escuela de Nutrición, Universidad de la República, Montevideo, Uruguay; ^7^Studies Coordinating Centre, Research Unit Hypertension and Cardiovascular Epidemiology, KU Leuven Department of Cardiovascular Sciences, University of Leuven, Leuven, Belgium; ^8^Cardiovascular Research Institute (CARIM), Maastricht University, Maastricht, Netherlands

## Abstract

Many public health policies in Latin America target an optimized sodium and potassium intake. The aims of this study were to assess the sodium and potassium intake using 24-hour urinary analysis and to study their association with blood pressure in a Uruguayan population cohort using cluster analysis. A total of 149 participants (aged 20–85 years) were included in the study, and office blood pressure, anthropometric measurements, biochemical parameters in the blood, and 24-hour urine samples were obtained. The overall mean sodium and potassium excretion was 152.9 ± 57.3 mmol/day (8.9 ± 3.4 g/day of salt) and 55.4 ± 19.6 mmol/day, respectively. The average office systolic/diastolic blood pressure was 124.6 ± 16.7/79.3 ± 9.9 mmHg. Three compact spherical clusters were defined in untreated participants based on predetermined attributes, including blood pressure, age, and sodium and potassium excretion. The major characteristics of the three clusters were (1) high systolic blood pressure and moderate sodium excretion, (2) moderate systolic blood pressure and very high sodium excretion, and (3) low systolic blood pressure and low sodium excretion. Participants in cluster three had systolic blood pressure values that were 23.9 mmHg (95% confidence interval: −29.5 to −1.84) lower than those in cluster one. Participants in cluster two had blood pressure levels similar to those in cluster one (*P* = 0.32) and worse metabolic profiles than those in cluster one and three (*P* < 0.05). None of the clusters showed high blood pressure levels and high sodium excretion. No linear association was found between blood pressure and urinary sodium excretion (r < 0.14;* P* > 0.47). An effect of sodium and potassium intake on blood pressure levels was not found at the population level using regression or cluster analysis.

## 1. Introduction

Cardiovascular disease is a major health problem that is strongly related to population growth and aging. High blood pressure (BP) remains the leading global risk factor for cardiovascular disease, and the highest BP levels have shifted from high-income to low-income countries during the past four decades [1]. In southern Latin America, the prevalence of hypertension is high [2]. Analysis of the effect of dietary sodium on BP and cardiovascular events has shown inconsistent results [3–5], and the optimal sodium intake for cardiovascular health remains under debate [6]. The average global sodium intake among adults has remained stable over the years at ~4000 mg/day (~10 g/day of salt) [7]. As a strategy to reduce population BP levels and prevent cardiovascular disease, the World Health Organization has recommended a sodium intake of <2000 mg/day (equivalent to 5 g/day of salt). At the same time, potassium intake was shown to be inversely correlated to BP levels [4] and has recently received interest as it may attenuate the harmful effect of high sodium levels on BP [8]. Despite most populations exhibiting high sodium intake, only a few develop hypertension, which demonstrates an individual susceptibility to sodium based on genetic and environmental backgrounds. The diverse clinical manifestations to sodium intake may indicate the existence of different BP phenotypes. Cluster analysis may assist in the identification of these BP phenotypes and provide an insight to better understand the complex relationship between BP and sodium and potassium excretion in real-life settings. This approach clusters individuals with homogeneous characteristics that can be further used as independent variables to study their relationship with health variables or outcomes. Therefore, this study aimed to assess the baseline status of sodium and potassium intake in a Uruguayan population cohort using 24-hour urinary analysis and to assess their association with BP using cluster analysis.

## 2. Materials and Methods

### 2.1. Study Design

The Genotipo Fenotipo y Ambiente de la Hipertension en Uruguay (GEFA-HT-UY) study is a prospective cohort that began recruiting in April 2012. The ethics committee of the University Hospital approved the study protocol, and all participants gave informed written consent. Detailed information about the study was published elsewhere [9]. Briefly, nuclear families were randomly recruited from the inhabitants of a geographically-defined area located approximately 10 km from downtown Montevideo. The cohort included family members older than 18 years and without an upper age limit.

During home visits, trained observers administered a standardized questionnaire inquiring into each participant's medical history, smoking, and drinking habits and medication intake. Examinations were undertaken at a health center located within the neighborhood. For the present analysis, 390 subjects were recruited, and the participation rate was 72.7%.

### 2.2. Anthropometric Measurements

Trained technicians measured body height to the nearest 0.5 cm using a pliable measurer (Seca, Germany) with the participant standing against a wall. Body weight measurements (HBF 415, Omron, Japan) were made to the nearest 100 grams with the participant wearing light indoor clothing without shoes. Body mass index (BMI) was calculated as weight (kg) divided by height (m^2^). Based on BMI, participants were classified as normal (18.5–24.9 kg/m^2^), overweight (25.0–29.9 kg/m^2^), or obese (≥30 kg/m^2^). Underweight category (<18.5 kg/m^2^) was excluded from the analysis, due to the small number of subjects (n = 2).

Waist and hip circumference measurements were made using an inelastic measuring tape (Seca, Germany). The waist-to-hip ratio was calculated as waist circumference (cm) divided by hip circumference (cm).

### 2.3. BP Measurements

BP was measured according to European guidelines [10]. After participants had rested for 5 minutes in a sitting position, trained observers obtained five consecutive BP readings (phase I systolic pressure and phase V diastolic pressure) to the nearest 2 mmHg using a mercury sphygmomanometer. Standard cuffs had a 12 x 24 cm inflatable portion; however, if the upper arm girth exceeded 31 cm, larger cuffs (15 x 35 cm bladders) were used. The five BP readings were averaged for the analysis. Participants were classified as hypertensive when office systolic BP (SBP) was at least 140 mmHg, office diastolic BP (DBP) was at least 90 mmHg, or antihypertensive drugs were being used.

### 2.4. Blood Samples

Venous blood samples were obtained in the morning after 12-hour fasting and were kept at 4°C. Within a 2-hour period, biochemical analysis of serum creatinine, cholesterol, and glucose levels was performed. Diabetes mellitus was fasting glucose level of ≥126 mg/dL (7 mmol/L) or use of antidiabetic drugs.

### 2.5. 24-Hour Urinary Collection

All participants were instructed (using cartographic and written instructions) to collect a 24-hour urine sample using a wide-neck bottle and were requested to report the first and last urination times. Urine samples could be collected during working days or at weekends. Female participants were instructed to perform the collection during nonmenstrual days.

The 24-hour urine collection protocol is difficult and cumbersome; therefore various completeness levels of the urine assessment method were proposed to avoid over- and undercollection. In this study, 24-hour urine collection was considered valid when the collection length was at least 18 hours, the urinary volume was >499 mL/day, and expected-to-observed 24-hour creatinine ratio was ≥0.6 [11].

Urinary metabolite and total urine volume values were corrected to a 24-hour period. After applying the abovementioned criteria and excluding participants with extreme sodium or potassium excretion (n = 10), only 149 participants had a valid 24-hour urine collection.

The 24-hour urinary sodium and potassium excretion values were used as surrogates for the daily sodium and potassium intake. Salt (NaCl) consumption was estimated using the 24-hour urinary sodium excretion. The conversion from sodium to NaCl was calculated using NaCl (g) = sodium (g) × 2.54.

### 2.6. Laboratory Methods

Urinary samples were processed in a certified laboratory. Urinary sodium and potassium levels were determined using the ion-selective electrode method (Cobas 6000, Roche, Switzerland). Daily urinary sodium, potassium, urea, and creatinine excretion were calculated as the product of urine metabolite concentration by urine volume. The sodium to potassium ratio was computed as sodium (mEq/day) divided by potassium (mEq/day). Serum creatinine was measured using the kinetic Jaffé method with the modifications described elsewhere [12]. A calibration standard traceable to an isotope dilution mass spectrometry (IDMS) was used according to the current recommendations [13].

### 2.7. Statistical Analysis

SAS software version 9.4 (SAS Institute, Cary, NC, USA) was used for database management and statistical analysis, and R software Core Team-2017 (R Foundation for Statistical Computing, Vienna, Austria) was used for the cluster analysis.

Continuous variables were expressed as the mean ± standard deviation (SD), and categorical variables were expressed as absolute number and proportions. The characteristics of participants were assessed using tertiles of the urinary sodium excretion distribution. Means and proportions were compared using a Student's t-test (or ANOVA) and a Chi-squared test, respectively.

Considering that only part of the population exhibits salt sensitivity and that hypertension is a complex phenotype, cluster analysis was performed to group participants into clusters. Standardized SBP, age, and sodium and potassium urinary excretion were used as predetermined attributes. Briefly, this method searches for groups of individuals called clusters having simultaneously the lowest variability within clusters and the greatest variability between clusters across SBP, age, sodium, and potassium variables. The clusters were found using hierarchical cluster, based on Ward algorithm which verifies that the ratio between the variability within the clusters versus the variability between clusters is the largest. The number of clusters set by this criteria aims to find compact spherical clusters [14]. Each individual was then assigned to one of the derived clusters.

Cluster analysis was employed using two approaches: including all participants and including only those not receiving antihypertensive treatment. After the clustering method was applied, a four-cluster solution was chosen based on dendrogram examination. When considering only the participants not using antihypertensive treatment, a three-cluster solution was chosen (see Figure S1 in the Supplementary Material). The differences in BP predictors were assessed between the clusters by comparing the minimum, mean, median, and maximum for the independent variable in each cluster. Associations between the abovementioned models and other variables were studied using the Chi-squared test. Linear regression models were also performed with standardized explanatory variables (i.e., age, sex, and sodium and potassium excretion) and including cluster groups as the dummy variable (cluster one as a reference).

To describe cluster characteristics we considered variable values between -0.5 and 0.5 SD as moderate, between -0.5 to -1.0 SD and 0.5 to 1.0 SD as low and high, respectively, and lower/higher than -1.0/1.0 as very low/very high attribute. Subsequently, the association between BP and 24-hour urinary sodium (and 24-hour urinary potassium) was assessed by computing Pearson's correlation coefficients. SBP and DBP were used as the response variables in linear regression models, and urinary sodium and potassium excretion, age, and sex were the explanatory variables.

Statistical significance was considered when* P* < 0.05.

## 3. Results

### 3.1. Characteristics of Participants

This analysis included 89 women (59.7%) with an average age (±SD) of 52.7 ± 16.5 years (range: 20.3–85.5 years). Of the 149 participants, 54 (36.2%) were hypertensive, 14 (9.4%) had diabetes, 30 (20.1%) were current smokers, and 59 (39.6%) reported drinking alcohol at least once a week. Of the hypertensive patients, 39 (72.2%) were receiving antihypertensive treatment.

The average participant weight was 74.1 ± 14.8 kg, height was 163 ± 11 cm, waist-to-hip ratio was 0.90 ± 0.08, fasting glucose was 94.6 ± 20.3 mg/dL, total cholesterol was 211.5 ± 41.0 mg/dL, and estimated glomerular filtration rate was 93.9 ± 20.7 mL/min/1.73 m^2^. The average office SBP/DBP was 124.6 ± 16.7/79.3 ± 9.9 mmHg. Women had lower weight, height, BMI, and waist-to-hip ratio (*P *< 0.0001); lower DBP (*P *= 0.052); lower urinary sodium, potassium, and creatinine excretion (*P *< 0.01); and lower regular alcohol intake (*P *< 0.01) compared to men.

Table 1 provides the characteristics of the participants by tertiles of sodium excretion. Anthropometric parameters (*P *< 0.001) and the triglyceride-to-high-density lipoproteins (HDLc) ratio (*P *< 0.05) but not BP (*P *> 0.15) increased with urinary sodium excretion.

### 3.2. Urinary Sodium Excretion

The overall 24-hour urinary sodium excretion was 152.9 ± 57.3 mmol/day (8.9 ± 3.4g/day of NaCl). Sodium excretion was >2.0 g/day in 89.9% of participants. On average, urinary sodium excretion was similar regardless of an individual's BP status. Nevertheless, when adjusting urinary sodium excretion by body weight, normotensive participants had higher sodium excretion levels than hypertensive participants (*P *< 0.01; Table 2). The estimated daily urinary sodium excretion was higher in men than in women with an average difference of 35.2 mmol (95% confidence interval (CI): 17.0–53.3;* P *< 0.001); however, after adjusting for body weight, this sex difference was lost (*P *= 0.95; Table 2). Sodium excretion increased with BMI (*P *< 0.0001 for trend); however, after adjusting the urinary sodium excretion for body weight, the relationship was lost (*P *= 0.13 for trend). Participants in the fourth age quartile had significantly lower urinary sodium excretion levels compared to the youngest participants (*P *= 0.02).

### 3.3. Urinary Potassium Excretion

The overall mean potassium intake estimated from the 24-hour urinary excretion was 55.4 ± 19.6 mmol (2166 ± 649 mg/day). Most individuals (95.3%; 60.6% of women and 39.4% of men) had an estimated potassium intake <3.5 g/day. Estimates in men were 10.1 mmol higher than in women (95% CI: 3.9–16.4;* P*<0.01). Potassium excretion was similar in hypertensive and normotensive individuals even after weighting potassium excretion for the glomerular filtration rate (Table 2). Daily urinary potassium excretion increased from normal weight to overweight (*P *< 0.001) but did not change between overweight and obese (*P *= 0.83; Table 2).

### 3.4. BP, Age, and Sodium and Potassium Clusters

SBP and DBP tended to be higher (*P *> 0.20) in subjects with sodium excretion >2.0 g/day (~5 g salt) compared to those with lower urinary excretion (see Table S1 in the Supplementary Material).

Cluster analysis was performed, firstly using the whole population divided into four BP clusters (Table 3A). Regression modeling showed a difference in SBP between clusters one and three. Participants in cluster three had average SBP values that were 1.37 ± 0.18 SD lower than participants in cluster one (R^2^ = 0.62;* P *≤ 0.0001). No difference was observed in sodium or potassium excretion.  Table S2 in the Supplementary Material summarizes the characteristics of participants by clusters.

Considering the influence of drug treatment on BP levels, further cluster analysis was performed excluding those participants receiving antihypertensive treatment (n = 39). Three BP clusters were derived, and the composition and relationship of the original variables and the clusters were determined. Cluster one grouped those with high SBP and a moderate sodium-excretion phenotype and was characterized by older participants with moderate potassium excretion, cluster two grouped those with moderate SBP levels and a very high sodium-excretion phenotype and was characterized by young subjects with high levels of potassium excretion, and cluster three grouped those with low SBP and low sodium excretion phenotype and was characterized by young participants with a below-average potassium excretion phenotype (Table 3B). Clusters were similar in terms of the sex composition, smoking and drinking habits of participants, and prevalence of diabetes.

In this group of participants without antihypertensive treatment, linear regression showed that women had lower SBP than men by an average of 4.8 mmHg (95% CI: −8.8 to −6.9;* P = *0.022). Regression modeling illustrated a difference in SBP between clusters one and three (Table 3B). Participants in cluster three (Table 3B) had an average SBP value that was 23.9 mmHg (95% CI: −29.5 to −1.84) lower than those in cluster one (*P *< 0.0001).  Table 4 shows the distribution of characteristics and the mean variable values within the clusters. Cluster one was represented by older participants (*P *< 0.0001) with higher waist-to-hip ratio (*P *= 0.004), BP (*P *< 0.0001), fasting glucose (*P *= 0.0041), and electrolyte excretion (*P *< 0.05) values and lower heart rate (*P *= 0.036) and eGFR (*P *< 0.0001) than cluster three participants. A higher prevalence of hypertension was also found in cluster one. Clusters two and three were represented by younger individuals (*P *> 0.05); nevertheless, participants from cluster two had higher BMI and waist-to-hip ratio values (*P *< 0.0001), higher fasting glucose and triglyceride-to-HDLc ratio values (*P *< 0.01), and higher BP and urinary electrolyte excretion values (*P *< 0.0001).

Furthermore, SBP and DBP were not lineally correlated with urinary sodium excretion after age and sex were included as covariates (r ≤ 0.19;* P *≥ 0.046). Consistently, urinary sodium excretion did not predict SBP or DBP using linear regression.

### 3.5. Urea Excretion and Urinary Osmolality

Urinary urea excretion did not differ with BP status; however, it was higher in men and showed a positive tendency with BMI (Table 2). Urinary osmolality was higher in men than women and increased across BMI categories (*P *< 0.0001 for trend) (Table 2). Those participants with sodium excretion above recommendation had higher (*P *< 0.05) urinary osmolality and urea excretion (see Table S1 in the Supplementary Material).

## 4. Discussion

In this population cohort, a high proportion of participants (98.3% of men and 84.3% of women) showed sodium intake that was over the current recommendations as assessed through 24-hour urinary sodium excretion values [15]. The mean daily sodium excretion in our study (152.9 ± 57.3 mmol/day) was below the global assessment values (161.8 mmol, 95% CI: 156.6–168.8 mmol/day and 171.8 mmol, 95% CI: 169.2–174.4 mmol/day) recently reported based on 24-hour urinary excretion [7, 16]. Global estimates for South America (including Argentina, Brazil, Chile, and Colombia) based on spot morning urine samples (204.4 ± 62.2 mmol) were also higher than the estimates obtained here [17]. The daily average sodium excretion was also reported by other Brazilian (180.9 ± 71.3 mmol) [18], Chilean (194.9 ± 80.0 mmol) [19], and Argentinian (203.6 ± 59.2 mmol) [17] studies. Conversely, the findings obtained here were closer to those recently reported in the United States (156.9 mmol; 95% CI: 148.5–165.4) [20], Canada (144.6 ± 63.8 mmol) [21], England (136 ± 61.3 mmol) [22], and Mexico (137.0 mmol; 95% CI: 132.8–141.2) [23].

In line with previously published estimates [20], urinary sodium excretion in this cohort was higher in men than women (*P *< 0.001); however, in contrast to previous reports, hypertensive participants had lower urinary sodium excretion than normotensive participants, although the difference was not significant [18, 24]. The fact that hypertensives had a tendency to lower urinary sodium excretion may be related to medical and health counseling that recommends that they reduce their sodium consumption in adjunct to hypertensive drug treatment.

The prevalence of overweight and obese participants in this cohort (69.8%; 95% CI: 62%–77%) was similar to national estimates (64.7%; 95% CI: 62.2%–67.3%). These individuals had higher sodium excretion levels than those with a normal body weight, which could be related to the fact that people with excess body weight tend to have higher energy intake which may be indicative of higher sodium consumption. Recent publications suggest that high sodium intake could be a risk factor for obesity independently of energy intake [25].

Only 6.7% of men and 3.4% of women in this study reached the currently recommended potassium consumption as assessed through their 24-hour urinary potassium excretion. The average estimate of urinary potassium excretion was similar to that reported for South America (53.5 ± 14.8 mmol/day) [17] and the United States (55.1 mmol; 95% CI: 51.9–58.3) [20]. The mean sodium to potassium urinary ratio in this study was 2.9 ± 1.2, which exceeds the current recommendation that considers that this ratio should be < 1.0.

**Figure 1:**
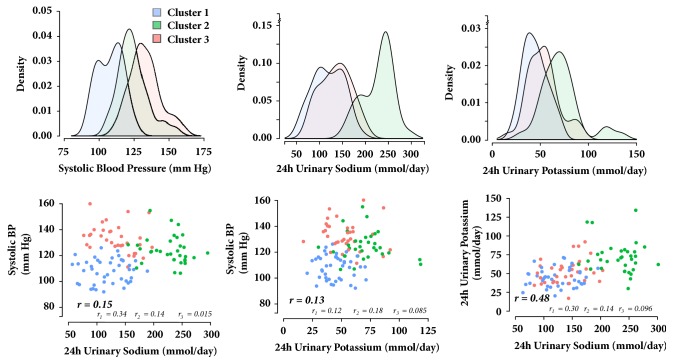
Density of systolic blood pressure (a), 24-hour urinary sodium (b), and potassium (c) excretion by cluster. Correlations between 24-hour urinary sodium and office systolic blood pressure (d) and 24-hour urinary potassium excretion and office systolic blood pressure (e), and the correlation between 24-hour urinary excretion of sodium and potassium (f). *r*: correlation coefficients.

The cluster analysis approach was useful for showing the complexity of establishing an association between a behavioral attribute (i.e., an isolated factor in the diet, such as sodium and potassium intake) and a characteristic phenotype (i.e., BP levels). Further, through cluster analysis none of the clusters showed high sodium excretion and high BP levels (see Figure 1). Many possible explanations can be considered. As the BP responses to sodium reduction may be diverse, the high sodium–high BP group might have been underrepresented in this study. Cluster one grouped participants with higher BP levels regardless of them demonstrating moderate sodium excretion levels. This could be associated with them being a “salt-sensitive” group; however, this condition was not assessed. Nevertheless, the proportion of individuals that display salt-sensitive BP changes is variable and ranges from 25%–50% in normotensive individuals to 40%–75% among hypertensive individuals [26], older people, or subjects with renal disease. Participants in cluster one had the highest prevalence of hypertension and were the oldest. On the other hand, sodium sensitivity is less frequently seen in white people, and this cohort included mostly white participants.

Clusters two and three were characterized by younger participants with different BP behavior. Participants in cluster two, regardless of their higher sodium excretion rates, seemed to have attenuated BP levels may be due to a protective effect of high potassium excretion, as was documented previously [8]. Moreover, as mentioned previously, the BP behavior observed in cluster two may be related to the fact that only a small proportion of the population is considered salt sensitive; therefore the high sodium excretion in this group may have a lower effect on SBP. Participants in cluster two showed a worse metabolic profile than those in cluster three, as they had a higher BMI, waist-to-hip ratio, fasting glucose level, and triglyceride-to-HDLc ratio (*P *< 0.0001). These parameters were established as clinical predictors associated with salt sensitivity and increased cardiovascular risk associated with sodium retention [27].

A direct relationship between BP levels and sodium excretion was reported in randomized clinical trials on salt intake [28–30] and in observational studies [3, 4]; however, most studies showed a small magnitude of effect, which was smaller in normotensive than hypertensive participants. An estimate from a large longitudinal population-based study showed a 1.7 mmHg increase in SBP per 100 mmol increase in 24-hour urinary sodium excretion [3]. Furthermore, a recent meta-analysis [31] reported a 1% decrease in BP when normotensive individuals reduced from a normal (≥150 mmol) to a subnormal (<120 mmol) level of sodium consumption in (SBP: −1.27 mmHg; 95% CI: −1.88 to −0.66;* P *= 0.0001 and DBP: −0.05 mmHg; 95% CI: −0.51 to 0.42;* P *= 0.85). Nevertheless, regardless of the reported increase in SBP associated with higher sodium excretion, it did not translate into a greater risk of developing hypertension in relation to sodium excretion (Hazard ratio: 0.98; 95% CI: 0.86–1.12 for participants in the high-sodium excretion group) [3].

In hypertensive participants, decreasing the sodium intake to a subnormal level caused a 3.5% reduction in BP (SBP: −5.48 mmHg; 95% CI: −6.53 to −4.43;* P *< 0.00001 and DBP: −2.75 mmHg; 95% CI: −3.34 to −2.17;* P *< 0.00001) [31]. A similar reduction in SBP (−5.39 mmHg; 95% CI: −6.62 to −4.15) was recently reported in another meta-analysis [32].

Besides the established relationship between BP and urinary electrolyte, in this population cohort, no linear association was found between BP and sodium excretion after adjustment for factors known to be associated to BP. The analyses that excluded participants under treatment were confirmatory; however, they were performed with even fewer subjects. This lack of association between BP and urinary sodium excretion may be explained by the modest described effect of salt intake on BP levels at a population level [3] and the low number of subjects in our study that completed a valid 24-hour urinary collection. In these sense, the relation between BP and sodium excretion in this population sample may have not been evident.

Some limitations of this study need to be addressed. First, the estimation of sodium consumption through urinary excretion may be underestimated by a single 24-hour urine collection. Titze et al. [33] showed that a single 24-hour urine collection was not sufficient due to the day-to-day variability. Moreover, cross-sectional association with BP could be underestimated; therefore accurate estimates should include multiple 24-hour urinary collections. However, the 24-hour urine collection is more accurate than sodium and potassium estimation from spot urine samples using the Kawasaki formula. Rigorous procedures were used to validate urine collection, leading therefore to a small sample size selection of participants. Potassium excretion should also be considered due to its influence on BP [34]. Nevertheless, potassium excretion measured by 24-hour urinary excretion was low (<60 mmol/day) in this cohort.

Overall, these findings are in line with emerging evidence that shows that a steady state of sodium is achieved after weeks and months of sodium accumulation and excretion, which occur independently of salt intake as a consequence of immune system regulation and storage in the skin, subcutaneous lymphatic networks, and muscle [35]. These multiple physiological mechanisms that regulate the effects of sodium (including but not limited to BP) may partially explain these results.

The strengths of this study include the validated urinary measurements of sodium and potassium excretion and the standardized BP measurement methods. Moreover, no data on salt and potassium consumption based on 24-hour urine collection have previously been reported in a healthy Uruguayan population.

In conclusion, the mean sodium excretion was higher than previously national-established data based on nutritional surveys. In untreated participants, three clusters were described based on BP, age, sodium intake, and potassium intake, which shows the complex mechanisms underlying BP regulation. No linear association was found between BP levels and electrolyte excretion in this population cohort. Therefore, these results could suggest that the magnitude of dietary effects on BP levels is poor in this Uruguayan population cohort. Sodium intake and potassium intake should probably not be considered as isolated factors but should be viewed in the context of physiological mechanisms that regulate BP (i.e., the renin-angiotensin system or sympathetic nervous system) and the effects of metabolism on sodium and BP regulation.

## Figures and Tables

**Table 1:** Characteristics of participants by tertiles of urinary sodium excretion.

**Variable **	**Tertile 1 **	**Tertile 2 **	**Tertile 3 **	***P***
	**(N=49) **	**(N=49) **	**(N=51)**	
**Number (%) with characteristic **				
Current smoking	14 (28.6)	8 (16.3)	8 (15.7)	0.23
Drinking alcohol	16 (32.7)	20 (40.8)	23 (45.1)	0.32
Hypertension	23 (46.9)	15 (30.6)	16 (31.4)	0.16
On antihypertensive drugs	17 (73.9)	12 (80.0)	10 (62.5)	0.22
Diabetes mellitus	3 (6.1)	3 (6.1)	8 (15.7)	0.16
**Mean (SD) characteristic **				
Age (y)	56.1±17.7	52.2±16.9	49.9±14.6	0.16
Height (cm)	159.5±8.0	164.9±13.1	167.2±8.7	0.0008
Weight (kg)	64.8±11.6	73.7±13.9	83.4±12.7	<.0001
Body mass index (kg/m^2^)	25.5±4.1	27.3±5.2	29.8±4.2	<.0001
Waist-to-hip ratio	0.86±0.068	0.91±0.074	0.93±0.081	<.0001
Office blood pressure				
Systolic pressure (mm Hg)	125.1±21.1	122.3±16.3	126.1±11.7	0.51
Diastolic pressure (mm Hg)	78.4±9.4	77.8±9.3	81.4±10.7	0.15
Heart rate (beats per minute)	71.1±8.2	71.3±11.3	71.3±9.4	0.99
UNa (mmol/day)	92.2±17.5	143.9±13.8	219.8±31.7	<.0001
UK (mmol/day)	44.0±12.1	54.3±18.6	67.3±19.8	<.0001
Fasting glucose (mg/dL)	92.0±14.2	95.3±28.6	96.5±15.0	0.53
Serum cholesterol (mg/dL)	223.1±46.2	200.2±37.4	211.4±36.5	0.022
Triglyceride to HDLc ratio	2.61±1.80	2.73±2.13	3.88±3.13	0.018
eGFR (mL/min/1.73 m2)	88.5±22.4	95.9±20.2	97.0±19.1	0.09

UNa: urinary sodium excretion; HDLc: high density lipoprotein cholesterol. Hypertension was a blood pressure of at least 140 mm Hg systolic or 90 mm Hg diastolic or use of antihypertensive drugs. Diabetes mellitus was a self-reported diagnosis, a fasting plasma glucose of 126 mg/dL or higher, or use of antidiabetic drugs. Estimated glomerular filtration rate (eGFR) was derived from the Chronic Kidney Disease Epidemiology Collaboration equation. *P* value for trend across tertiles of urinary sodium excretion.

**Table 2:** Sensitivity analysis according to BMI, age quartiles, sex, blood pressure status, and eGFR.

**Mean (SD) characteristic**	**N**	**UNa (mmol/d)**	**UNa-BW (mmol/kg/d)**	**Na/Cr ratio**	**UK (mmol/d)**	**K/Cr ratio**	**Urinary volume (L/24-h)**	**Urinary urea excretion (g/d)**	**Urinary Osmolality*♯* (mOsm/kg)**
All	149	152.9±57.3	2.07±0.70	2.62±0.95	55.4±19.6	1.61±0.51	1.56±0.64	22.3±8.5	417.3±136.8
BMI (Kg/m^2^)									
Normal	43	117.8±43.3^§^	1.94±0.68	2.44±0.86	46.6±12.2^†^	1.67±0.55	1.41±0.57	17.9±6.3^‡^	327.0±73.4^§^
Overweight	66	158.3±51.9^§^	2.10±0.66	2.67±1.01	59.7±23.0^†^	1.67±0.55	1.63±6.93	23.2±9.9^‡^	426.5±138.1^§^
Obesity	38	183.3±62.0^§^	2.08±0.65	2.69±0.94	58.8±16.2^†^	1.46±0.34	1.65±5.87	26.1±6.2^‡^	496.6±137.2^§^
Age (y)									
< 41	42	165.1±57.6^*∗*^	2.23±0.73	2.59±1.05	55.8±22.1	1.44±0.42^*∗*^	1.41±4.97	23.5±9.1	448.4±136.6
≥ 41 < 58	42	157.3±62.4^*∗*^	2.15±0.77	2.75±0.96	51.1±16.7	1.56±0.52^*∗*^	1.62±7.95	21.1±8.0	420.4±144.1
≥ 58 < 68	36	149.2±57.8^*∗*^	1.91±0.66	2.53±0.95	59.5±21.0	1.72±0.59^*∗*^	1.62±6.25	24.8±9.0	427.2±144.3
≥ 68	29	133.5±44.4^*∗*^	1.94±0.55	2.59±0.80	55.6±17.4	1.80±0.44^*∗*^	1.64±5.69	19.6±7.2	367.8±110.9
Women	89	138.7±56.9^‡^	2.08±0.78	2.78±1.07^†^	51.3±17.7^†^	1.75±0.55^§^	1.53±6.51	19.6±7.4^§^	372.9±122.6^§^
Men	60	173.9±51.7^‡^	2.07±0.56	2.39±0.69^†^	61.4±20.9^†^	1.42±0.37^§^	1.61±6.20	26.3±8.6^§^	481.6±132.1^§^
Hypertensive	54	143.2±55.9	1.87±0.60^†^	2.54±0.88	55.1±18.4	1.65±0.44	1.57±5.98	21.9±8.2	398.5±125.6
Normotensive	95	158.4±57.7	2.19±0.73^†^	2.67±0.98	55.5±20.3	1.59±0.55	1.56±6.63	22.6±8.8	428.7±143.0
eGFR ≤ 60	9	119.0±54.2	1.78±0.61	2.57±1.01	46.0±22.0	1.59±0.32	1.40±5.98	17.3±5.6	340.1±149.2
eGFR > 60	140	155.1±57.0	2.09±0.70	2.62±0.95	56.0±19.4	1.61±0.52	1.57±6.41	22.8±8.6	424.1±134.4

BMI: body mass index, UNa: urinary sodium excretion, UNa-BW: urinary sodium excretion adjusted by body weight, Na/Cr: urinary sodium to creatinine ratio, UK: urinary potassium excretion, K/Cr: urinary potassium to creatinine ratio, eGFR: estimated glomerular filtration rate in mL/min/1.73 m2. *♯*Urinary osmolality was calculated excluding participants under diuretics drugs (n=2), as follows: 2[Na(mmol) + K(mmol)] + urea(mg/dL)/2.8. *P* values are for comparisons within each column and represent the results of contrasting two categories (sex, blood pressure status and eGFR) or trend when more than two categories were represented (BMI and age): ^*∗*^*P* ≤ 0.05; ^†^*P* ≤ 0.01; ^‡^*P* ≤ 0.001; ^§^*P* ≤ 0.0001.

**Table 3:** Standardized means of age, systolic blood pressure, urinary sodium, and potassium*∗* excretion by cluster in whole sample (A; n=149) and in participants not using antihypertensive treatment (B; n=110).

	**A**				**B**		
	**Cluster 1**	**Cluster 2**	**Cluster 3**	**Cluster 4**	**Cluster 1**	**Cluster 2**	**Cluster 3**
	**N=47**	**N=23**	**N=34**	**N=45**	**N=34**	**N=34**	**N=42**
**Mean (SD) characteristic**							
Age (y)	**0.74±0.68**	0.71±0.62	-0.75±0.91	-0.57±0.67	**0.85±0.76**	-0.32±0.75	-0.43±0.93
Systolic pressure (mm Hg)	**0.64±0.90**	0.58±0.87	-1.11±0.53	-0.12±0.59	**0.82±0.76**	0.24±0.73	-0.86±0.62
UNa (mmol/day)	-0.70±0.53	0.42±0.94	-0.66±0.54	**1.01±0.74**	-0.39±0.58	**1.21±0.57**	-0.66±0.58
UK (mmol/day)	-0.52±0.60	**1.50±0.92**	-0.62±0.63	0.24±0.67	-0.12±0.81	**0.79±1.05**	-0.55±0.63

*∗*Bold denotes higher value within the cluster. A cluster represents a group of individuals with similar systolic blood pressure values, age, and urinary sodium and potassium excretion

**Table 4:** Characteristics of participants (n=110) by cluster.

**Variable**	**Cluster 1 **	**Cluster 2 **	**Cluster 3**	***P value***
	**N=34**	**N=34**	**N=42**	*(Chi* ^*2*^ or* ANOVA)*
**Number (%) with characteristic**				
Female	21 (61.8)	16 (47.1)	29 (69.0)	0.15
Current smoking	7 (6.4)	6 (5.5)	12 (10.9)	0.31
Drinking alcohol	14 (12.7)	13 (11.8)	16 (14.5)	0.95
Hypertension	9 (8.2)	5 (4.5)	1 (0.9)	0.0095
Diabetes mellitus	3 (2.7)	1 (0.9)	0 (0)	0.11
**Mean (SD) characteristic **				
Age (y)	62.7±12.4*∗*	43.5±12.3	41.7±15.1	<.0001
Height (cm)	162.1±9.9	167.6±8.6	164.8±12.6	0.11
Weight (kg)	69.6±14.1	83.6±11.1	68.0±13.3	<.0001
Body mass index (kg/m^2^)	26.5±4.5	29.8±3.6	25.1±5.1	<.0001
Waist-to-hip ratio	0.90±0.069*∗*	0.92±0.072	0.85±0.082	0.0002
Office blood pressure				
Systolic pressure (mm Hg)	132.7±11.1*∗*	124.1±10.8	107.9±9.1	<.0001
Diastolic pressure (mm Hg)	81.8±8.0*∗*	82.2±9.9	72.0±7.2	<.0001
Heart rate (beats per minute)	68.9±12.0*∗*	71.2±7.0	73.9±9.0	0.11
Fasting glucose (mg/dL)	94.7±17.3*∗*	95.3±12.5	85.9±7.0	0.0023
Serum cholesterol (mg/dL)	218.7±45.2	213.8±34.3	201.5±39.3	0.16
Triglyceride to HDLc ratio	2.80±1.67	3.94±3.14	2.02±1.28	0.0009
UNa (mmol/day)	135.2±33.5*∗*	227.6±32.8	119.2±33.6	<.0001
UK (mmol/day)	53.1±16.2*∗*	71.4±21.1	44.6±12.6	<.0001
Sodium to potassium ratio	2.77±1.22	3.48±1.27	2.88±1.13	0.033
Urinary creatinine excretion (g/day)	1.25±0.36	1.79±0.57	1.26±0.40	<.0001
Urinary urea excretion (g/day)	19.0±7.5	30.7±8.7	19.1±5.7	<.0001
eGFR (mL/min/1.73 m2)	87.7±13.6*∗*	105.0±16.6	103.2±18.6	<.0001

Hypertension was a blood pressure of at least 140 mm Hg systolic or 90 mm Hg diastolic, or use of antihypertensive drugs. Diabetes mellitus was a self-reported diagnosis, a fasting plasma glucose of 126 mg/dL or higher, or use of antidiabetic drugs. Estimated glomerular filtration rate (eGFR) was derived from the Chronic Kidney Disease Epidemiology Collaboration equation. *∗P*<0.05 value for difference between clusters 1 and 3.

## Data Availability

All data were generated for this article. Datasets are not available for public domain according to national legislation and may be available upon request to corresponding author.

## Supplementary Materials

Supplementary Materials Table S1. Blood pressure, urine osmolality, and urea excretion according to levels of sodium intake within or above recommendation. Table S2. Characteristics of participants (n=149) by cluster. Figure S1. Dendrogram of hierarchical cluster analysis using 24-hour urinary sodium and potassium excretion, age, and office systolic blood pressure.
