# Supplementary material for: Blood Pressure in relation to 24-Hour Urinary Sodium and Potassium Excretion in a Uruguayan Population Sample
Source: Int J Hypertens. 2018 Dec 2;2018:6956078. doi: 10.1155/2018/6956078 (PMC6304641; doi:10.1155/2018/6956078)

SUPPLEMENTARY MATERIAL

Blood pressure in relation to 24-hour urinary sodium and potassium excretion in a Uruguayan population sample

Paula Moliterno, Ramón Álvarez-Vaz, Matias Pécora, Leonella Luzardo, Luciana Borgarello, Alicia Olascoaga, Carmen Marino Donangelo, Oscar Noboa, Jan A. Staessen, and José Boggia*.

*Correspondance to:

Email: jboggia@hc.edu.uy

Number of Tables 2 Figures 1

Correspondence should be addressed to:
José Boggia MD, PhD
Centro de Nefrología y Depto de Fisiopatología
Av.Italia 2870, Piso 14 Ala Oeste. PC 11600
Montevideo, Uruguay

Cel:+598 9919 1146   Fax +598 24809850
Email: jboggia@hc.edu.uy

Table S1: Blood pressure, urine osmolality and urea excretion according to levels of sodium intake within or above recommendation

**Mean (SD) characteristic**	**< 2.0 g/day**	**≥ 2.0 g/day**	***P***
Office BP (mm Hg)			
Systolic BP	114.5±12.6	121.1±14.8	0.20
Diastolic BP	78.7±6.7	78.1±9.9	0.87
Urinary Osmolality (Mosm/Kg)	231.9±41.5	437.2±129.7	<0.0001
Urinary urea excretion (g/day)	13.5±4.6	23.3± 8.8	0.016
Urine Volume (L/24-h)	1.27±0.71	1.54±0.64	0.22

Estimates and SD express variables associated to levels of sodium intake in the whole population excluding those under hypertension treatment (n=9; n=101). To convert sodium from mg to mmol divide by 22.99.

Table S2: Characteristics of participants (n=149) by blood pressure cluster

Variable	Cluster 1<br>N=47	Cluster 2<br>N=23	Cluster 3<br>N=34	Cluster 4<br>N=45	***P value***<br>*(Chi*^{2} or *ANOVA)*
Number (%) with characteristic					
Female	33 (22.2)	8 (5.4)	25 (16.8)	23 (15.4)	0.0068
Current smoking	11 (7.4)	0 (0)	9 (6.0)	10 (6.7)	0.048
Drinking alcohol	18 (12.1)	9 (6.0)	13 (8.7)	19 (12.8)	0.83
Hypertension	26 (17.5)	14 (9.4)	4 (2.7)	10 (6.7)	<0.0001
On antihypertensive drugs	19 (73.0)	11 (78.6)	3 (75.0)	6 (60.0)	0.0002
Diabetes mellitus	5 (3.4)	6 (4.0)	0 (0)	3 (2.0)	0.0094
Mean (SD) characteristic					
Age (y)	64.9±11.2*	64.4±10.3	40.3±15.0	43.3±11.1	<0.0001
Height (cm)	159.8±9.4	167.7±10.0	163.7±13.2	166.4±8.4	0.0045
Weight (kg)	67.7±12.5	81.9±12.1	66.3±12.0	82.6±14.1	<0.0001
Body mass index (kg/m2)	26.5±4.1	29.0±2.7	25.0±5.3	29.8±4.9	<0.0001
Waist-to-hip ratio	0.90±0.065*	0.95±0.075	0.84±0.075	0.92±0.075	<0.0001
Office blood pressure					
Systolic pressure (mm Hg)	135.2±15.1*	134.2±14.5	106.0±8.8	122.5±9.9	<0.0001
Diastolic pressure (mm Hg)	82.5±8.7*	82.0±9.6	71.3±7.3	80.5±9.9	<0.0001
Heart rate (beats per minute)	69.5±9.9	69.9±12.1	73.9±9.8	71.9±7.4	0.24
Fasting glucose (mg/dL)	99.9±30.9*	99.6±15.2	86.2±6.8	92.7±11.4	0.013
Serum cholesterol (mg/dL)	219.0±42.6	225.0±44.5	204.1±40.0	202.2±35.7	0.056
Triglyceride to HDLc ratio	2.99±1.87	3.08±2.52	2.01±1.22	3.98±3.32	0.0059
UNa (mmol/day)	112.9±30.6	177.1±45.6	115.3±31.0	210.8±42.2	<0.0001
UK (mmol/day)	45.2±11.8	84.7±18.0	43.2±12.4	60.2±13.1	<0.0001
Sodium to potassium ratio	2.66±1.13	2.16±0.69	2.92±1.22	3.65±1.11	<0.0001
Urinary creatinine excretion (mg/day)	1152.0±321.4	1729.3±543.4	1189.7±350.9	1641.3±478.1	<0.0001
Urinary urea excretion (g/day)	18.3±7.1	29.6±9.5	18.2±5.4	25.9±6.9	<0.0001
eGFR (mL/min/1.73 m2)	83.1±20.1*	82.1±17.3	104.7±15.0	103.1±18.2	<0.0001

Abbreviations: HDLc (high density lipoprotein cholesterol), UNa (urinary sodium excretion), UK (urinary potassium excretion), eGFR (estimated glomerular filtration rate). Hypertension was a blood pressure of at least 140 mm Hg systolic or 90 mm Hg diastolic, or use of antihypertensive drugs. Diabetes mellitus was a self-reported diagnosis, a fasting plasma glucose of 126 mg/dL or higher, or use of antidiabetic drugs. eGFR was derived from the Chronic Kidney Disease Epidemiology Collaboration equation. *P<0.01 for difference between cluster 1 and 3.

Figure S1: Dendrogram of hierarchical cluster analysis using 24-hour urinary sodium and potassium excretion, age and office systolic blood pressure.

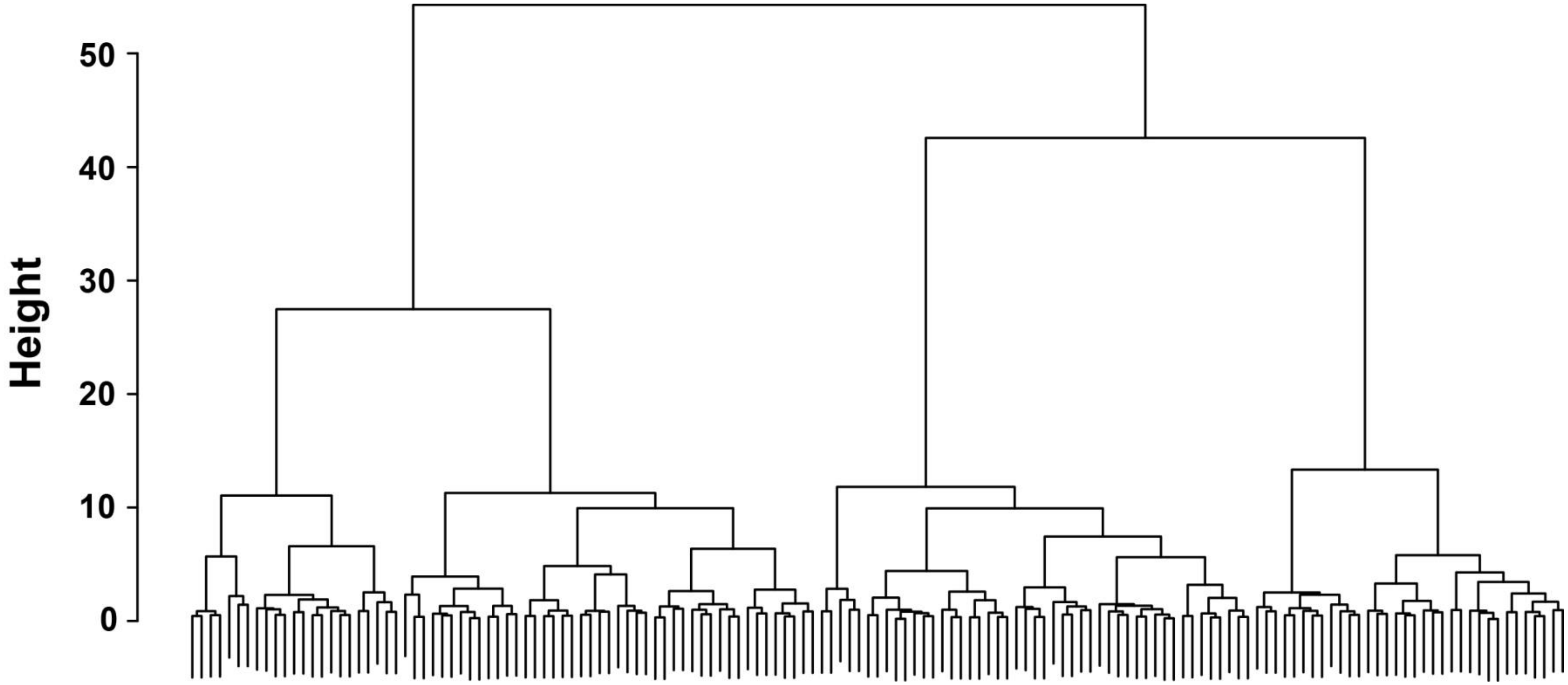

Supplement: Supplementary Materials — Table S1. Blood pressure, urine osmolality, and urea excretion according to levels of sodium intake within or above recommendation. Table S2. Characteristics of participants (n=149) by cluster. Figure S1. Dendrogram of hierarchical cluster analysis using 24-hour urinary sodium and potassium excretion, age, and office systolic blood pressure. [file 6956078.f1.pdf]
